# Predictors of 90-Day Mortality and the Association of Reperfusion Therapy with 90-Day Mortality in Intermediate-High and High-Risk Pulmonary Embolism: A Real-World Multidisciplinary Cohort Study

**DOI:** 10.3390/life16071189

**Published:** 2026-07-17

**Authors:** Özgür Batum, Merve Ayık Türk, Yelda Varol, Muhammed Emin Arslan, Cenk Sarı, Sami Deniz, Nigar Dirican, Kutluhan Eren Hazır, Sibel Doruk

**Affiliations:** 1 Department of Pulmonology, University of Health Sciences Türkiye, Izmir Faculty of Medicine, Izmir City Hospital, Izmir 35353, Türkiye; ozgurbat@yahoo.com (Ö.B.); yeldavatansever@hotmail.com (Y.V.); arslanemin@hotmail.com (M.E.A.); sami_deniz@yahoo.com (S.D.); 2 Department of Cardiology, University of Health Sciences Türkiye, Izmir Faculty of Medicine, Izmir City Hospital, Izmir 35540, Türkiye; drcenksari@gmail.com (C.S.);; 3 Department of Pulmonology, University of Health Sciences Izmir City Hospital, Izmir 35540, Türkiye; nigardirican@yahoo.com (N.D.); sibeldoruk@yahoo.com (S.D.)

**Keywords:** pulmonary embolism, thrombolytic therapy, catheterization, mortality, risk assessment

## Abstract

**Background:** Intermediate-high and high-risk pulmonary embolism (PE) are associated with substantial mortality despite anticoagulation. While reperfusion therapies may improve outcomes, real-world evidence comparing treatment strategies and identifying predictors of mid-term mortality remains limited. We aimed to evaluate predictors of 90-day mortality and the impact of reperfusion therapy in patients with intermediate-high and high-risk PE managed within a multidisciplinary Pulmonary Embolism Response Team (PERT). **Methods:** This retrospective cohort study included 114 consecutive patients with intermediate-high or high-risk acute PE admitted to a tertiary referral center between October 2023 and December 2024. Risk stratification was performed according to ESC guidelines, and simplified Pulmonary Embolism Severity Index (sPESI) scores were calculated. Treatment strategies included anticoagulation alone or reperfusion therapy (systemic thrombolysis or catheter-directed therapy). The primary endpoint was all-cause 90-day mortality. Multivariate logistic regression was performed to identify independent predictors of mortality. **Results:** The mean age was 68 ± 16 years, and 88% had at least one comorbidity. Reperfusion therapy was administered to 27% of patients. Mortality rates were 1% at 24 h, 6% at 7 days, 17% at 30 days, and 25% at 90 days. In multivariate analysis, hemoglobin ≤ 11.7 g/dL (OR 5.06, 95% CI 1.58–16.17, *p* = 0.006), sPESI > 2 (OR 4.86, 95% CI 1.49–15.84, *p* = 0.009), prior stroke (OR 11.59, 95% CI 2.08–64.51, *p* = 0.005), and high 30-day mortality risk classification (OR 14.14, 95% CI 1.19–167.89, *p* = 0.036) were independent predictors of mortality. Reperfusion therapy was independently associated with a significant reduction in 90-day mortality (OR 0.05, 95% CI 0.003–0.65, *p* = 0.022). The regression model demonstrated good explanatory power (Nagelkerke R^2^ = 0.487). **Conclusions:** In this real-world cohort of intermediate-high and high-risk PE, reperfusion therapy was independently associated with lower 90-day mortality and low bleeding risk. In addition to established predictors, sPESI retained strong prognostic value beyond its traditional 30-day timeframe. These findings support risk-adapted reperfusion strategies guided by multidisciplinary teams and reinforce the role of integrated clinical risk assessment in optimizing outcomes.

## 1. Introduction

Acute pulmonary embolism (PE) remains a major cause of cardiovascular morbidity and mortality worldwide and is the third leading cause of cardiovascular death after myocardial infarction and stroke [1]. Clinical presentation ranges from asymptomatic cases to cardiogenic shock and death, with prognosis largely determined by hemodynamic status, right ventricular dysfunction, and comorbid conditions. Early risk stratification is therefore essential for guiding therapeutic decisions and improving outcomes. Patients classified as high-risk or intermediate-high-risk represent a particularly vulnerable subgroup, with significantly increased mortality despite prompt initiation of anticoagulation therapy [1,2].

Systemic thrombolysis is strongly recommended for high-risk PE because of its ability to rapidly restore pulmonary perfusion, reduce right ventricular afterload, and improve survival [1]. In contrast, the optimal management of intermediate-high-risk PE remains controversial. Although anticoagulation alone is the standard initial treatment, this subgroup carries a substantial risk of early clinical deterioration and death. Several randomized trials and observational studies, including SEATTLE II and ULTIMA, have demonstrated that catheter-directed thrombolysis and other reperfusion therapies can significantly improve right ventricular function and pulmonary hemodynamics [3,4,5]. The PEERLESS randomized controlled trial (2024) further compared large-bore mechanical thrombectomy with catheter-directed thrombolysis in intermediate-high-risk PE, evaluating clinical improvement and complication rates [6]. Furthermore, recent meta-analyses have suggested that reperfusion therapies may reduce mortality compared with anticoagulation alone while maintaining acceptable safety profiles [7,8]. However, despite these promising findings, current international guidelines do not recommend routine use of systemic thrombolysis in intermediate-high-risk PE due to concerns regarding major bleeding complications, including intracranial hemorrhage [1,9]. As a result, clinicians often face uncertainty when selecting reperfusion strategies in this population, and treatment decisions frequently rely on individualized risk-benefit assessment rather than definitive guideline-based recommendations. The implementation of multidisciplinary Pulmonary Embolism Response Teams (PERTs) has emerged as an important strategy to facilitate individualized decision-making and optimize patient selection for reperfusion therapy in real-world practice [10].

In addition to treatment selection, accurate risk stratification tools are critical for identifying patients at highest risk of adverse outcomes. The simplified Pulmonary Embolism Severity Index (sPESI) is a validated and guideline-recommended clinical score widely used to predict 30-day mortality in acute PE [1,11]. Multiple validation studies have confirmed its utility in identifying patients at low or high risk of early mortality and guiding initial management [11,12]. However, although sPESI was originally developed to predict short-term outcomes, its prognostic value beyond 30 days remains incompletely defined. Emerging evidence suggests that baseline risk stratification scores may retain prognostic significance beyond the acute phase, but data specifically evaluating predictors of mid-term mortality, such as 90-day outcomes, in intermediate-high and high-risk PE patients—particularly in cohorts managed with contemporary reperfusion strategies—remain limited.

Several clinical, laboratory, and imaging parameters have consistently been associated with mortality in patients with acute pulmonary embolism. Clinical risk stratification tools, particularly the simplified Pulmonary Embolism Severity Index (sPESI), remain the most widely validated predictors of short-term mortality and are recommended by current international guidelines for initial risk assessment [1,11,12]. In addition, hemodynamic instability, right ventricular dysfunction, elevated cardiac biomarkers (troponin and natriuretic peptides), renal dysfunction, anemia, advanced age, and a high burden of comorbidities have repeatedly been identified as adverse prognostic factors [1,2]. Nevertheless, most available evidence has focused on early (30-day) mortality or unselected acute PE populations. Whether these established predictors retain similar prognostic significance for mid-term (90-day) mortality, particularly among intermediate-high- and high-risk patients managed within multidisciplinary Pulmonary Embolism Response Team (PERT) programs and contemporary reperfusion strategies, remains insufficiently investigated.

The aim of the study was to identify independent predictors of 90-day mortality and to evaluate whether reperfusion therapy was independently associated with 90-day mortality after adjustment for clinically relevant covariates in intermediate-high- and high-risk pulmonary embolism managed within a contemporary multidisciplinary PERT program.

## 2. Material and Methods

### 2.1. Study Design and Setting

This retrospective cohort study was conducted at Izmir City Hospital, a tertiary referral center specializing in pulmonary and cardiovascular diseases. The hospital serves as the largest regional referral center for pulmonary embolism (PE) management and operates a multidisciplinary Pulmonary Embolism Response Team (PERT) consisting of pulmonologists, cardiologists, and intensive care specialists, with access to interventional radiology and cardiovascular surgery when required. Every patient presenting to the emergency department with suspected or confirmed PE is evaluated by the PERT at admission, and treatment decisions are made by consensus.

Consecutive adult patients diagnosed with acute pulmonary embolism between 10 October 2023, and 31 December 2024, were screened for eligibility.

### 2.2. Study Population

Patients were eligible for inclusion if they:

- were ≥18 years of age,
- had a confirmed diagnosis of acute pulmonary embolism,
- were classified as intermediate-high or high-risk PE according to the ESC Guidelines [1],
- and had complete clinical, laboratory, treatment, and follow-up data.

The diagnosis of pulmonary embolism was confirmed by computed tomographic pulmonary angiography (CTPA) or ventilation/perfusion (V/Q) scintigraphy.

Patients were excluded if they:

- had low-risk or intermediate-low-risk PE,
- had suspected or confirmed chronic thromboembolic pulmonary hypertension (CTEPH),
- had incomplete clinical or follow-up data,
- or were lost to follow-up before 90 days.

### 2.3. Risk Stratification

Risk stratification was based on 30-day mortality risk in accordance with the ESC Guidelines [1]. High-risk PE was defined as the presence of sustained hypotension (systolic blood pressure < 90 mmHg for ≥15 min), cardiogenic shock, syncope, or resuscitated cardiac arrest. Intermediate-high-risk PE was defined as normotensive PE with evidence of right ventricular dysfunction (on echocardiography or CT) and elevated cardiac biomarkers (troponin, NT-proBNP). The simplified Pulmonary Embolism Severity Index (sPESI) was calculated for each patient to further quantify predicted 30-day mortality risk [11].

### 2.4. Treatment Modalities

Treatment decisions were made by the multidisciplinary PERT based on clinical status, bleeding risk, and contraindications.

Patients received one of the following treatment strategies:

Catheter-directed thrombolysis (continuous rt-PA infusion into the pulmonary artery for 24 h) or ultrasound-assisted thrombolysis (EkoSonic system enhancing fibrin penetration);

Systemic thrombolysis (100 mg rt-PA intravenously over 2 h); or

Anticoagulation alone with intravenous unfractionated heparin (80 IU/kg bolus followed by 18 IU/kg/h infusion, adjusted to maintain aPTT 46–70 s) or subcutaneous low-molecular-weight heparin at 1 mg/kg every 12 h. Maintenance anticoagulation included warfarin, low-molecular-weight heparin, or direct oral anticoagulants, based on clinical judgment.

Patients initially treated with anticoagulation who subsequently required escalation to reperfusion therapy due to clinical deterioration were classified as treatment switch cases.

### 2.5. Data Collection

Collected data included demographic features such as age, sex, and smoking history, as well as comorbidities. Clinical information included pulmonary embolism risk factors and the presence of a genetic predisposition. At the time of diagnosis, echocardiographic findings were recorded in addition to baseline systolic pulmonary artery pressure (sPAP) and the Pulmonary Embolism Severity Index (PESI) or simplified PESI (sPESI) [11]. Laboratory data were systematically collected from the patient’s initial admission workup. Routine biochemical analyses, including brain natriuretic peptide (BNP) and high-sensitivity cardiac troponin, were performed in the hospital’s central biochemistry laboratory using standardized automated immunoassay techniques. All measurements were conducted in accordance with the manufacturer’s instructions as part of routine clinical practice, and the results were retrieved from the institutional electronic medical record system. D-dimer levels were measured at baseline for diagnostic confirmation but were not used in the follow-up evaluation. Further data included the presence and reasons for thrombolysis contraindications, treatment modality administered, hospital length of stay, and any requirement for treatment escalation.

If a patient who was initially managed with anticoagulation during the first two weeks subsequently required transition to thrombolytic therapy, this was documented as a treatment switch. In addition, because our hospital functions as a regional referral center, some patients were transferred from outside facilities where they had initially been managed with anticoagulation. In these cases, transition to thrombolysis was undertaken either due to clinical deterioration during follow-up or because the complexity of the case required multidisciplinary management. Similarly, patients who were initially classified as low- or intermediate-low-risk but later progressed to high- or intermediate-high-risk categories and received thrombolytic therapy were also recorded as treatment switch cases.

Transthoracic echocardiography was performed in all patients during hospitalization and follow-up by experienced cardiologists who were blinded to the clinical data. Examinations were carried out using a GE Vivid S60 ultrasound system (GE Healthcare, Chicago, IL, USA) equipped with a 3Sc-D phased-array transducer. Echocardiographic assessments were performed in accordance with the ESC Guidelines for Acute PE [1].

Systolic pulmonary artery pressure (sPAP) was estimated from the tricuspid regurgitant jet velocity using the modified Bernoulli equation (RVSP = 4 V^2^ + estimated right atrial pressure), where right atrial pressure was derived from inferior vena cava (IVC) diameter and its respiratory variation. In the absence of right ventricular outflow tract obstruction, right ventricular systolic pressure (RVSP) was considered equivalent to sPAP.

The following echocardiographic parameters were systematically evaluated: right ventricular (RV) enlargement in the parasternal long-axis view; RV/left ventricular (LV) basal diameter ratio; interventricular septal flattening (“D-shaped” left ventricle); IVC dilatation with reduced respiratory variability; the 60/60 sign (RV outflow tract velocity < 60 cm/s and tricuspid regurgitation gradient < 60 mmHg); tricuspid annular plane systolic excursion (TAPSE); and tricuspid annular systolic velocity (S′). These parameters were used to assess RV size, systolic function, and pressure load as part of the standardized echocardiographic protocol applied to all study participants.

The primary outcome of the study was all-cause mortality at 90 days following the diagnosis of acute PE. Secondary outcomes included all-cause mortality at 24 h, 7 days, and 30 days, as well as in-hospital mortality and bleeding complications.

Bleeding events were classified as major or minor according to standardized definitions. Major bleeding was defined in accordance with the Bleeding Academic Research Consortium (BARC) criteria [13]. Minor bleeding was defined as any clinically overt bleeding requiring medical evaluation or intervention that did not meet the criteria for major bleeding.

The primary outcome was all-cause mortality within 90 days after the diagnosis of pulmonary embolism. Mortality status at 90 days was determined using hospital electronic medical records and verified through follow-up records and the national health registry when available.

### 2.6. Statistical Analysis

Statistical analyses were performed using SPSS version 23.0 (IBM Corp., Armonk, NY, USA). Continuous variables were tested for normality using the Kolmogorov–Smirnov test. A priori sample size estimation was performed using G*Power 3.1.9.7 (Heinrich Heine University Düsseldorf, Düsseldorf, Germany) based on the effect size of 0.303, α = 0.05, power 80%, a minimum sample size of 104 patients. Normally distributed variables were expressed as mean ± standard deviation (SD) and compared using Student’s t-test, while non-normally distributed variables were presented as median [interquartile range, IQR] and compared with the Mann–Whitney U-Test. Categorical variables were expressed as numbers and percentages and compared using the Chi-Square Test or Fisher’s Exact Test, as appropriate. ROC curve analysis was performed to determine optimal cutoff values for continuous variables associated with 90-day mortality. Univariable Cox proportional hazards analyses were explored as complementary time-to-event analyses. However, because of the limited number of mortality events and very small event counts in several subgroups, multivariable Cox regression was not performed to avoid model instability and overfitting. Univariate logistic regression analysis was performed to identify factors associated with 90-day mortality. Variables with *p* < 0.10 in the univariate analysis were considered eligible for multivariable modeling. Variable selection was guided primarily by clinical relevance and biological plausibility, together with the results of univariable analyses, rather than by automated stepwise procedures alone. To reduce the risk of overfitting, only variables considered clinically relevant and not demonstrating substantial collinearity were retained in the final model. The primary endpoint was predefined as all-cause mortality within 90 days after acute PE diagnosis. Because the study objective was to identify baseline predictors of mortality status at this fixed time point, multivariable logistic regression was selected as the primary analytical method. Mortality status at 90 days was available for all included patients, and patients lost to follow-up before 90 days were excluded; therefore, censoring was not considered a major analytical issue in the primary analysis. Mortality status was recorded descriptively, and mortality was additionally reported at 24 h, 7 days, and 30 days to characterize the temporal distribution of deaths. Odds ratios (ORs) and 95% confidence intervals (CIs) were calculated. Model performance was assessed using the Nagelkerke R^2^ and Hosmer-Lemeshow goodness-of-fit test. A two-sided *p* < 0.05 was considered statistically significant.

## 3. Results

### 3.1. Baseline Characteristics

A total of 114 patients with intermediate-high or high-risk pulmonary embolism were included in the study. The mean age was 68 ± 16 years (range 22–97), and 62 patients (54%) were female. The mean body mass index was 29.2 ± 6.2 kg/m^2^. Nearly half of the cohort had a history of smoking, with a mean cumulative exposure of 35 ± 28 pack-years among smokers.

Comorbidities were highly prevalent, affecting 88% of patients. The most common comorbid conditions were hypertension (62%), diabetes mellitus (36%), and coronary artery disease or heart failure (36%). The mean length of hospital stay was 14 ± 16 days, and the mean follow-up duration was 274 ± 197 days (Table 1).

The most frequent provoking factors for pulmonary embolism were prolonged immobilization (26%) and recent surgery (25%) (Supplementary Table S1).

### 3.2. Risk Stratification and Clinical Characteristics

According to ESC risk classification, 98 patients (86%) were categorized as intermediate-high-risk and 16 patients (14%) as high-risk pulmonary embolism. The median sPESI score was 2 (range 0–5), with the majority of patients having scores ≥2. Right ventricular dilatation was present in all patients at diagnosis.

Laboratory findings demonstrated a median D-dimer level of 109 μg/L (range 7–5403) and a median NT-proBNP level of 437 pg/mL (range 15–35,000) (Supplementary Table S2).

### 3.3. Treatment Strategies and Bleeding Outcomes

All patients received anticoagulation therapy, and 31 patients (27%) underwent reperfusion therapy. Systemic thrombolysis was administered in 22 patients (19%), catheter-directed therapy in 1 patient (1%), and ultrasound-assisted thrombolysis in 8 patients 7%.

Among high-risk patients, 56.2% received systemic thrombolysis and 6.2% received catheter-directed therapy, whereas only 37.5% were treated with anticoagulation alone. In contrast, the majority of intermediate-high-risk patients were managed with anticoagulation 78.6%, while 13.3% received systemic thrombolysis and 8.2% underwent catheter-directed therapy (Supplementary Table S3).

When stratified by baseline ESC risk category, reperfusion therapy was associated with lower 90-day mortality in both intermediate-high and high-risk groups. Among intermediate-high-risk patients, 90-day mortality was 27.3% with anticoagulation versus 0.0% with reperfusion therapy (*p* = 0.005). Among high-risk patients, 90-day mortality was 83.3% with anticoagulation versus 30.0% with reperfusion therapy (*p* = 0.119), although statistical power was limited by small sample size (Supplementary Table S4).

Intensive care unit admission was required in 68 patients (60%). Thrombolysis was contraindicated in 23 patients (20%), most commonly due to recent surgery or bleeding risk.

Bleeding complications occurred in 9 patients (8%). Major bleeding was observed in only 2 patients (2%), while minor bleeding occurred in 7 patients (6%). Baseline characteristics stratified according to treatment strategy are presented in Table 2.

### 3.4. Mortality Outcomes

Mortality increased over time: 1% at 24 h, 6% at 7 days, 17% at 30 days, and 25% at 90 days. The in-hospital mortality was 16%, and the overall mortality at follow-up was 29%. When stratified according to treatment strategy, the 90-day mortality rate was 31.3% (26/83) in the anticoagulation group, 13.6% (3/22) in the systemic thrombolysis group, and 0.0% (0/9) in the catheter-directed therapy group (*p* = 0.045).

### 3.5. Comparison of Survivors and Non-Survivors

Univariate comparison of baseline characteristics between survivors and non-survivors demonstrated several significant differences.

Non-survivors were significantly older than survivors (median 78 vs. 70 years, *p* = 0.007). They also had significantly lower hemoglobin levels (median 10.9 vs. 12.4 g/dL, *p* = 0.003), higher sPESI scores (median 3 vs. 2, *p* < 0.001), and greater cumulative smoking exposure (median 40 vs. 28 pack-years, *p* = 0.029). These findings indicate that advanced age, anemia, higher clinical severity, and greater smoking burden were associated with increased mortality risk.

No significant differences were observed between the two groups in D-dimer levels, platelet counts, creatinine levels, or liver function parameters, suggesting that baseline coagulation markers and organ function tests were not independently associated with mortality in this cohort (Table 3).

### 3.6. ROC Curve Analysis

Receiver operating characteristic (ROC) curve analysis was performed to evaluate the discriminatory performance of clinical and laboratory variables for predicting 90-day mortality.

Among the evaluated variables, sPESI demonstrated the strongest predictive performance, with an area under the curve (AUC) of 0.744 (95% CI 0.654–0.821, *p* < 0.001), indicating good discriminatory ability. The optimal cutoff value was >2, yielding a sensitivity of 55% and specificity of 85%, confirming its utility in identifying patients at increased risk of mortality. Hemoglobin also demonstrated significant predictive value, with an AUC of 0.685 (95% CI 0.591–0.769, *p* = 0.001), reflecting moderate discriminatory performance. The optimal cutoff value was ≤11.7 g/dL, consistent with its independent association with mortality observed in multivariate analysis. Age and cumulative smoking exposure also showed moderate predictive ability, although their discriminatory performance was inferior to that of sPESI (Supplementary Table S5). Overall, these findings confirm that sPESI is the most robust clinical predictor of 90-day mortality among evaluated variables.

### 3.7. Univariate Predictors of 90-Day Mortality

Univariate logistic regression analysis identified several clinical, laboratory, and treatment-related variables significantly associated with 90-day mortality.

Among demographic variables, age >66 years was associated with a markedly increased risk of mortality (OR 5.56, 95% CI 1.78–17.34, *p* = 0.001). Similarly, heavy smoking exposure (>47 pack-years) was significantly associated with mortality (OR 5.08, 95% CI 1.27–20.36, *p* = 0.016). Regarding comorbid conditions, prior cerebrovascular disease was a strong predictor of mortality (OR 5.09, 95% CI 1.47–17.61, *p* = 0.006), and chronic kidney disease was also significantly associated with increased risk (OR 3.67, 95% CI 1.23–10.93, *p* = 0.015). Among laboratory and risk stratification variables, hemoglobin ≤11.7 g/dL was associated with a more than five-fold increased mortality risk (OR 5.34, 95% CI 2.11–13.56, *p* < 0.001). Similarly, sPESI score > 2 was strongly associated with mortality, demonstrating the highest predictive value among clinical risk parameters (OR 6.82, 95% CI 2.66–17.46, *p* < 0.001).

Importantly, treatment strategy was also significantly associated with outcomes. Patients receiving reperfusion therapy had significantly lower odds of 90-day mortality than those treated with anticoagulation alone (OR 0.23, 95% CI 0.07–0.84, *p* = 0.018), demonstrating an independent association between reperfusion therapy and lower 90-day mortality (Table 4 and Table 5; Supplementary Tables S7 and S8).

### 3.8. Multivariate Predictors of 90-Day Mortality

Multivariate logistic regression analysis was performed to identify independent predictors of 90-day mortality. Variables with *p* < 0.10 in univariate analysis were included in the multivariate model.

Hemoglobin ≤11.7 g/dL remained independently associated with a significantly increased risk of mortality (OR 5.06, 95% CI 1.58–16.17, *p* = 0.006). Similarly, sPESI score > 2 was a strong independent predictor, confirming its prognostic value in identifying high-risk patients (OR 4.86, 95% CI 1.49–15.84, *p* = 0.009). History of cerebrovascular disease emerged as one of the strongest predictors, increasing mortality risk more than eleven-fold (OR 11.59, 95% CI 2.08–64.51, *p* = 0.005). In addition, patients classified as high-risk according to ESC risk stratification had significantly increased mortality risk compared with intermediate-high-risk patients (OR 14.14, 95% CI 1.19–167.89, *p* = 0.036). Importantly, reperfusion therapy was independently associated with a marked reduction in mortality risk (OR 0.05, 95% CI 0.003–0.65, *p* = 0.022), demonstrating a strong protective effect after adjustment for clinical severity and comorbidities.

The multivariate model demonstrated good explanatory power and calibration, with a Nagelkerke R^2^ of 0.487 and a non-significant Hosmer–Lemeshow test (*p* = 0.656), indicating good model fit (Table 6).

In an additional model incorporating treatment as reperfusion therapy (systemic thrombolysis or catheter-directed therapy) versus anticoagulation alone, reperfusion therapy remained independently associated with lower odds of 90-day mortality (OR 0.24, 95% CI 0.07–0.82, *p* = 0.028), supporting the consistency of the observed association across different model specifications.

## 4. Discussion

In this real-world cohort of patients with intermediate-high and high-risk pulmonary embolism, we identified several important findings. First, reperfusion therapy was independently associated with a marked reduction in 90-day mortality, even after adjustment for clinical severity and comorbid conditions. Second, established clinical risk markers, including anemia, prior cerebrovascular disease, and higher sPESI scores, were strong independent predictors of mortality. Third, the sPESI score demonstrated robust discriminatory ability for predicting not only short-term but also mid-term mortality, supporting its broader prognostic utility beyond the traditional 30-day timeframe. Together, these findings highlight the importance of integrated clinical risk stratification and suggest that reperfusion therapy may be independently associated with lower 90-day mortality in appropriately selected patients.

One of the most important findings of our study is the strong independent association between reperfusion therapy and improved mortality. However, given the observational design and non-randomized treatment allocation, this finding should be interpreted as an association rather than evidence of a causal treatment effect. After adjustment for clinical severity and comorbidities, reperfusion therapy remained independently associated with a marked reduction in 90-day mortality, supporting a true therapeutic benefit beyond baseline risk differences. Importantly, treatment allocation in our cohort reflected clinical severity, with reperfusion therapy more frequently administered to high-risk patients. Therefore, treatment decisions were based on multidisciplinary clinical judgment rather than random allocation, and the possibility of residual confounding and selection bias cannot be excluded despite multivariable adjustment. These findings are consistent with prior studies demonstrating the potential benefits of reperfusion strategies in intermediate-high and high-risk pulmonary embolism. The SEATTLE II and ULTIMA trials demonstrated significant improvements in right ventricular function and pulmonary hemodynamics with catheter-directed thrombolysis, with acceptable safety profiles [3,4]. Similarly, recent research has suggested that catheter-based reperfusion therapies may improve clinical outcomes and reduce mortality compared with anticoagulation alone in selected patients [7,8,14]. Although current ESC guidelines recommend systemic thrombolysis primarily for high-risk PE and do not support its routine use in intermediate-high-risk patients because of bleeding concerns, our findings provide real-world evidence suggesting that reperfusion therapy may be associated with lower 90-day mortality in carefully selected intermediate-high-risk patients [1].

Importantly, the association between reperfusion therapy and lower 90-day mortality remained evident across ESC-defined risk strata, particularly among patients with intermediate-high-risk pulmonary embolism. However, given the observational design of the study, this finding should be interpreted as an association after adjustment for measured clinical severity and comorbidities rather than evidence of a causal treatment effect. This finding is consistent with prior observational studies and registry data suggesting that carefully selected intermediate-high-risk patients may experience lower 90-day mortality following reperfusion therapy, particularly when treatment decisions are guided by clinical deterioration, right ventricular dysfunction, and multidisciplinary assessment [1,11,15,16]. Notably, these results are also aligned with the recently published 2026 AHA/ACC/ACCP/CHEST multisociety guideline, which emphasizes a risk-adapted treatment approach and supports consideration of reperfusion therapy in selected intermediate-high-risk patients with evidence of clinical vulnerability or impending hemodynamic compromise [17]. By demonstrating an independent association between reperfusion therapy and lower 90-day mortality, particularly among intermediate-high-risk patients, our findings provide contemporary real-world evidence supporting current guideline recommendations for individualized treatment escalation in carefully selected patients. Although subgroup inferences in the high-risk cohort remain limited by sample size, the overall consistency of the observed effect reinforces the potential role of carefully selected reperfusion therapy in improving outcomes beyond anticoagulation alone.

An important consideration when evaluating reperfusion therapy is the associated bleeding risk. In our cohort, the incidence of major bleeding was low (2%), which is notably lower than rates reported in previous trials and observational studies, where major bleeding rates typically range from 5% to 12% depending on patient selection and treatment modality [3,7,8]. This favorable safety profile may reflect careful patient selection, multidisciplinary decision-making, and close clinical monitoring within a PERT-based management framework. These findings suggest that, in appropriately selected patients, reperfusion therapy may be associated with lower 90-day mortality without a proportional increase in major bleeding events. Importantly, the low incidence of major bleeding was observed despite the high comorbidity burden and advanced age of our cohort, suggesting that careful risk stratification and multidisciplinary treatment selection may mitigate bleeding risk even in clinically vulnerable populations.

Our study confirms and extends the prognostic value of the simplified Pulmonary Embolism Severity Index (sPESI) in patients with intermediate-high and high-risk pulmonary embolism. Consistent with prior validation studies, higher sPESI scores were strongly associated with increased mortality risk [11,12]. Importantly, in our cohort, sPESI not only remained an independent predictor in multivariate analysis but also demonstrated the strongest discriminatory performance among evaluated clinical variables, highlighting its robustness as a prognostic tool.

Notably, sPESI demonstrated good discriminatory ability for predicting 90-day mortality, suggesting that its prognostic relevance extends beyond its originally intended 30-day prediction window. This finding is clinically important, as risk stratification tools are often used primarily to guide early management decisions, yet mortality risk in pulmonary embolism frequently extends beyond the acute phase. Our results indicate that sPESI captures not only acute hemodynamic compromise but also broader markers of physiological vulnerability that influence mid-term outcomes. These findings are supported by previous observational studies showing that baseline clinical severity indices remain predictive of mortality beyond the early phase of pulmonary embolism [18,19]. One possible explanation is that sPESI incorporates key clinical variables such as age, cardiopulmonary comorbidities, and markers of cardiovascular stress, which reflect both acute disease severity and underlying patient vulnerability. As a result, sPESI may serve as a composite indicator of overall physiological reserve, rather than solely reflecting the immediate hemodynamic impact of the embolic event.

In addition to embolism severity and treatment strategy, underlying comorbidities emerged as critical determinants of mortality risk in our cohort. Prior cerebrovascular disease was one of the strongest independent predictors of mortality, increasing the risk of death more than elevenfold. This finding is consistent with large registry-based studies demonstrating that patients with pre-existing cardiovascular and cerebrovascular disease represent a particularly vulnerable population with impaired physiological reserve and reduced tolerance to acute hemodynamic stress [1,11,12]. Chronic vascular disease is associated with endothelial dysfunction, impaired autoregulatory mechanisms, and reduced cardiopulmonary compensation capacity, all of which contribute to adverse outcomes following acute pulmonary embolism [20].

Chronic kidney disease was also independently associated with increased mortality risk in our study. Renal dysfunction has been consistently identified as an adverse prognostic factor in pulmonary embolism across multiple large cohort studies and registries [11,17]. The increased risk associated with renal impairment is likely multifactorial and includes chronic inflammation, endothelial dysfunction, increased thrombotic susceptibility, and impaired cardiopulmonary reserve. Furthermore, renal dysfunction may complicate clinical management by altering anticoagulant pharmacokinetics and increasing the risk of both thrombotic and bleeding complications [17].

Similarly, anemia emerged as an independent predictor of mortality. Previous studies have shown that anemia is associated with worse outcomes in acute PE, reflecting both reduced oxygen delivery capacity and increased overall comorbidity burden [21]. In the setting of acute pulmonary embolism, reduced hemoglobin levels may exacerbate right ventricular ischemia and impair systemic oxygen delivery, further compromising cardiopulmonary function during an already hemodynamically stressful event. In addition, anemia may serve as a marker of frailty and chronic disease, both of which are independently associated with worse clinical outcomes [18,21]. These observations underscore the importance of comprehensive risk assessment incorporating both acute disease severity and baseline patient characteristics. Identifying patients with increased comorbidity burden may facilitate individualized treatment decisions, closer monitoring, and optimized follow-up strategies, ultimately improving clinical outcomes.

Our findings support the importance of comprehensive risk stratification in patients with intermediate-high and high-risk pulmonary embolism. The strong prognostic performance of sPESI in predicting 90-day mortality suggests that its clinical utility extends beyond early risk assessment and may help identify patients who require closer monitoring and follow-up. In addition, the observed association between reperfusion therapy and lower 90-day mortality, without a substantial increase in major bleeding events, supports an individualized treatment approach guided by clinical severity and multidisciplinary evaluation. Current ESC guidelines recommend reperfusion therapy primarily for high-risk patients; however, our findings suggest that selected intermediate-high-risk patients may also benefit from carefully considered escalation of therapy [1]. These results reinforce the need for a personalized, risk-adapted approach that integrates clinical severity, comorbidity burden, and multidisciplinary assessment to optimize treatment selection and improve patient outcomes.

This study has several important strengths. First, it reflects real-world clinical practice in a high-volume tertiary referral center using a multidisciplinary PERT model, providing clinically relevant insights into contemporary management of intermediate-high and high-risk pulmonary embolism. Second, the study included a well-characterized cohort with comprehensive clinical, laboratory, and echocardiographic data, allowing detailed assessment of prognostic factors. Third, the use of multivariate regression analysis enabled the identification of independent predictors of mortality while accounting for potential confounding variables. Importantly, the study evaluated both early and mid-term mortality, providing additional prognostic information beyond the traditional 30-day timeframe. Finally, the integration of treatment strategy, clinical severity, and comorbidity burden allowed a comprehensive evaluation of factors influencing outcomes in this high-risk population.

Several limitations should be acknowledged. Firstly, the retrospective single-center design may limit the generalizability of the findings and introduce the potential for selection bias. Secondly, treatment allocation was not randomized and was based on clinical decision-making, which may introduce residual confounding despite multivariate adjustment. Thirdly, the relatively modest sample size may limit statistical power, particularly for subgroup analyses. In addition, the relatively limited number of mortality events compared with the number of variables included in the multivariable model may have increased the risk of model overfitting and contributed to the wide confidence intervals observed for several predictors. Although variable selection was restricted based on clinical relevance and univariate screening, the stability of the estimated odds ratios should be interpreted with caution. External validation in larger prospective cohorts is warranted. Fourthly, although follow-up was complete for mortality outcomes, other long-term clinical outcomes, such as recurrent embolism or functional status, were not systematically assessed. An additional limitation of the study was the inclusion of both intermediate-high-risk and high-risk pulmonary embolism patients within the primary analysis. Although both groups require close monitoring and may be candidates for reperfusion therapy, they differ substantially with respect to baseline prognosis and current guideline recommendations. Because the high-risk subgroup comprised only a small proportion of the study population, subgroup analyses were underpowered and should be interpreted cautiously. Consequently, the overall findings are largely driven by the intermediate-high-risk cohort and should not be interpreted as equally applicable to both risk categories. Another important limitation was the potential for confounding by indication. Because treatment allocation was based on multidisciplinary clinical judgment rather than randomization, patients selected for reperfusion therapy may have differed from those managed with anticoagulation alone in ways that could not be fully captured by measured variables. Although multivariable adjustment was performed using clinically relevant covariates, residual confounding cannot be excluded. Consequently, the observed association between reperfusion therapy and lower 90-day mortality should not be interpreted as evidence of a causal treatment effect and warrants confirmation in larger prospective studies using causal inference methods where appropriate. Finally, as an observational study, causal relationships between reperfusion therapy and improved mortality cannot be definitively established. Although multivariable adjustment was performed, residual confounding cannot be completely excluded because treatment allocation was based on multidisciplinary clinical judgment rather than randomization. Consequently, the observed association between reperfusion therapy and lower mortality should not be interpreted as proof of a causal treatment effect. Prospective randomized studies are required to confirm these findings.

## 5. Conclusions

In this real-world cohort, reperfusion therapy was independently associated with lower 90-day mortality after adjustment for measured confounders. However, because most patients were in the intermediate-high-risk category and the high-risk subgroup was relatively small, these findings should primarily be interpreted as reflecting the overall study cohort and require confirmation in larger studies specifically evaluating each risk category separately. In addition to established clinical predictors, sPESI demonstrated robust prognostic value beyond its traditional 30-day timeframe, supporting its utility in mid-term risk stratification. Furthermore, comorbid conditions such as cerebrovascular disease, chronic kidney disease, and anemia were important determinants of mortality, highlighting the role of underlying patient vulnerability. These findings support a personalized, risk-adapted approach integrating clinical severity, comorbidity assessment, and multidisciplinary decision-making to optimize treatment selection and improve outcomes. These results highlight the need for individualized, risk-adapted treatment strategies in acute PE and support the careful use of reperfusion therapy in selected populations.

## Figures and Tables

**Table 1:** Demographic and Clinical Characteristics of the Study Population.

Characteristic	Mean ± SD/n	%/Median (IQR)
**Total Number of Patients**	114	⸺
**Age, years**	68 ± 16	--
**Sex**		
Male	52	(46)
Female	62	(54)
**BMI, kg/m^2^**	--	28.1 (7.52)
**Smoking status**		
Current smoker	26	(23)
Former smoker	23	(20)
Never smoker	65	(57)
**Smoking exposure, pack-years (n = 49)**	--	30 (26.3)
**Length of hospital stay, days**	--	9 (7)
**Follow-up, days**	--	258 (266)
Presence of comorbidities	100	(88)
Hypertension	71	(62)
Diabetes mellitus	41	(36)
Heart failure/Coronary artery disease	41	(36)
Cerebrovascular event	12	(11)
Atrial fibrillation	18	(16)
Chronic kidney disease	16	(14)
Other comorbidities	61	(54)

Abbreviations: BMI, body mass index; SD, standard deviation. Normally distributed variables are presented as mean ± standard deviation, whereas non-normally distributed variables are presented as median (interquartile range).

**Table 2:** Treatment Characteristics and Complications of the Study Population.

Characteristic	N (140)	%
**ICU admission**	68	(60)
**Thrombolytic therapy**	31	(27)
**Thrombolysis method**		
Systemic	22	(19)
Catheter-directed	1	(1)
Ultrasound-assisted (EKOS)	8	(7)
Heparin/LMWH	83	(73)
**Contraindication to thrombolysis**	23	(20)
**Most frequent contraindications**		
Recent trauma/surgery (≤3 weeks)	10	(9)
Active bleeding/suspicion of bleeding	8	(7)
Stroke/intracranial pathology	5	(4)
**Maintenance therapy**		
Warfarin	56	(49)
LMWH	36	(32)
Rivaroxaban	22	(19)
**Treatment Switch**	21	(18)
**Bleeding complications**	9	(8)
**Bleeding severity**		
Minor	7	(6)
Major	2	(2)
**Bleeding site**		
Gastrointestinal	3	(3)
Other	3	(3)
Genitourinary	1	(1)
Pulmonary	1	(1)
Catheter-related	1	(1)

Abbreviations: ICU, intensive care unit; LMWH, low-molecular-weight heparin; EKOS, EkoSonic Endovascular System.

**Table 3:** Comparison of Patients According to 90-Day Mortality.

Characteristic	Total (n = 114) Median (Min–Max)	Deceased (n = 29) Median (Min–Max)	Survivors (n = 85) Median (Min–Max)	*p*-Value *
Age. years	72 (22–97)	78 (44–97)	70 (22–97)	**0.007**
BMI. kg/m^2^	28.1 (16.9–57.2)	26.7 (16.9–41.0)	28.6 (20.3–57.2)	0.058
Smoking. pack-years (n = 49)	30 (1–120)	40 (6–120)	28 (1–100)	**0.029**
D-dimer. μg/L	109 (7–5403)	110 (12–5403)	109 (7–2119)	0.616
sPAP. mmHg	45 (20–75)	45 (20–63)	45 (20–75)	0.481
Pro-BNP. pg/mL	437 (15–35,000)	5700 (130–35,000)	263 (15–35,000)	0.077
Hemoglobin. g/dL	12.1 (6.5–16.9)	10.9 (6.5–15.2)	12.4 (7.9–16.9)	**0.003**
Platelets. ×10^3^/µL	220 (30–629)	251 (30–629)	216 (100–500)	0.340
aPTT. seconds	27.45 (20.00–46.60)	28.40 (20.80–46.60)	27.40 (20.00–41.40)	0.283
Creatinine. mg/dL	1.03 (0.17–7.84)	1.10 (0.17–3.96)	0.99 (0.56–7.84)	0.541
ALT. U/L	23 (5–757)	23 (5–757)	23 (5–709)	0.349
sPESI	2 (0–5)	3 (1–5)	2 (0–4)	**<0.001**

* Mann–Whitney U Test. Abbreviations: BMI. body mass index; sPAP. systolic pulmonary artery pressure; Pro-BNP. pro–B-type natriuretic peptide; aPTT. Activated partial thromboplastin time; ALT. Alanine aminotransferase; sPESI. Simplified Pulmonary Embolism Severity Index.

**Table 4:** Clinical and Laboratory Parameters Associated with 90-Day Mortality.

Factor	Category	Mortality (+) (n = 29) n (%)	Mortality (−) (n = 85) n (%)	*p*-Value	OR (95% CI)
**Hemoglobin**	≤11.7 g/dL	21 (72%)	28 (33%)	**<0.001**	**5.34 (2.11–13.56)**
**sPESI score**	>2	16 (55%)	13 (15%)	**<0.001**	**6.82 (2.66–17.46)**
**30-day mortality risk**	High	8 (28%)	8 (9%)	**0.015**	**3.67 (1.23–10.93)**
**PE risk factor**	Present	14 (48%)	49 (58%)	0.381	0.69 (0.29–1.60)
**ICU admission**	Yes	19 (66%)	49 (58%)	0.456	1.40 (0.58–3.36)

OR: Odds Ratio; CI: Confidence Interval; sPESI: simplified Pulmonary Embolism Severity Index; ICU: intensive care unit; PE: pulmonary embolism.

**Table 5:** Analysis of Treatment Characteristics Associated with 90-Day Mortality.

Factor	Category	Mortality (+) (n = 29) n (%)	Mortality (–) (n = 85) n (%)	*p*-Value	OR (95% CI)
**Thrombolytic therapy**	Yes	3 (10%)	28 (33%)	**0.018**	**0.23 (0.07–0.84)**
**Maintenance therapy**	Warfarin or Rivaroxaban	13 (45%)	65 (76%)	**0.002**	**0.25 (0.10–0.61)**
	DMAH	16 (55%)	20 (24%)		Ref.
**Treatment Switch**	Yes	1 (3%)	20 (24%)	**0.016**	**0.12 (0.01–0.91)**
**Contraindication to thrombolysis**	Yes	5 (17%)	18 (21%)	0.648	0.78 (0.26–2.32)
**Bleeding complication**	Yes	0 (0%)	9 (11%)	0.068	-

OR: Odds Ratio; CI: Confidence Interval; LMWH: low-molecular-weight heparin.

**Table 6:** Multivariate Logistic Regression Analysis for 90-Day Mortality.

Variable	B	SE	*p*-Value	OR	95% CI
**Hemoglobin ≤ 11.7 g/dL**	1.620	0.593	**0.006**	**5.06**	1.58–16.17
**sPESI > 2**	1.582	0.602	**0.009**	**4.86**	1.49–15.84
**History of stroke**	2.450	0.876	**0.005**	**11.59**	2.08–64.51
**30-day mortality risk: High**	2.649	1.262	**0.036**	**14.14**	1.19–167.89
**Thrombolytic therapy**	−3.049	1.334	**0.022**	**0.05**	0.003–0.65
**Constant**	−2.694	0.554	<0.001	0.07	

Nagelkerke R^2^ = 0.487. Hosmer–Lemeshow test: *p* = 0.656. Abbreviations: SE. standard error; OR. odds ratio; CI. confidence interval; sPESI. Simplified Pulmonary Embolism Severity Index; CVE. cerebrovascular event.

## Data Availability

The datasets generated and/or analyzed during the current study are not publicly available due to institutional data protection policies but are available from the corresponding author on reasonable request.

## Supplementary Materials

The following supporting information can be downloaded at: https://www.mdpi.com/article/10.3390/life16071189/s1.

## Abbreviations

PE	Pulmonary Embolism
PERT	Pulmonary Embolism Response Team
ESC	European Society of Cardiology
sPESI	Simplified Pulmonary Embolism Severity Index
CTPA	Computed Tomography Pulmonary Angiography
V/Q	Ventilation/Perfusion
BNP	Brain Natriuretic Peptide
NT-proBNP	N-terminal pro-B-type Natriuretic Peptide
RV	Right Ventricle
LV	Left Ventricle
TAPSE	Tricuspid Annular Plane Systolic Excursion
sPAP	Systolic Pulmonary Artery Pressure
BARC	Bleeding Academic Research Consortium
OR	Odds Ratio
CI	Confidence Interval
SD	Standard Deviation
IQR	Interquartile Range

## References

[1] Konstantinides S.V., Meyer G., Becattini C., Bueno H., Geersing G.J., Harjola V.P., ESC Scientific Document Group (2019). 2019 ESC Guidelines for the diagnosis and management of acute pulmonary embolism developed in collaboration with the. European Respiratory Society (ERS): The Task Force for the diagnosis and management of acute pulmonary embolism of the European Society of Cardiology (ESC). Eur. Respir. J..

[2] Latsios G., Mantzouranis E., Kachrimanidis I., Theofilis P., Dardas S., Stroumpouli E., Aggeli C., Tsioufis C. (2025). Recent advances in risk stratification and treatment of acute pulmonary embolism. World J. Cardiol..

[3] Piazza G., Hohlfelder B., Jaff M.R., Ouriel K., Engelhardt T.C., Sterling K.M., Jones N.J., Gurley J.C., Bhatheja R., Kennedy R.J. (2015). SEATTLE II Investigators. A Prospective, Single-Arm, Multicenter Trial of Ultrasound-Facilitated, Catheter-Directed, Low-Dose Fibrinolysis for Acute Massive and Submassive. Pulmonary Embolism: The SEATTLE II Study. JACC Cardiovasc. Interv..

[4] Kucher N., Boekstegers P., Müller O.J., Kupatt C., Beyer-Westendorf J., Heitzer T., Tebbe U., Horstkotte J., Müller R., Blessing E. (2014). Randomized, controlled trial of ultrasound-assisted catheter-directed thrombolysis for acute intermediate-risk pulmonary embolism. Circulation.

[5] Lehr A., Guichet P., Garimella B., Krolikowski K., Amoroso N., Sista A., Brosnahan S.B. (2023). Impact of Time to Intervention on Catheter-Directed Therapy for Pulmonary Embolism. Crit. Care Explor..

[6] Giri J., Mahfoud F., Gebauer B., Andersen A., Friedman O., Gandhi R.T., Jaber W.A., Pereira K., West F.M. (2024). PEERLESS II: A Randomized Controlled Trial of Large-Bore Thrombectomy Versus Anticoagulation in Intermediate-Risk Pulmonary Embolism. J. Soc. Cardiovasc. Angiogr. Interv..

[7] Ismayl M., Machanahalli Balakrishna A., Aboeata A., Gupta T., Young M.N., Altin S.E., Aronow H.D., Goldsweig A.M. (2022). Meta-Analysis Comparing Catheter-Directed Thrombolysis Versus Systemic Anticoagulation Alone for Submassive Pulmonary Embolism. Am. J. Cardiol..

[8] Zoumpourlis P., Mangeshkar S., Chi K.Y., Varrias D., Spanos M., Fahimuddin M., Langston M.D., Khan U.A., Grushko M.J., Singh P. (2025). Catheter-Based Therapies in Acute Pulmonary Embolism-Mortality and Safety Outcomes: A Systematic Review and Meta-Analysis. J. Clin. Med..

[9] Harvey J.J., Huang S., Uberoi R. (2022). Catheter-directed therapies for the treatment of high risk (massive) and intermediate risk (submassive) acute pulmonary embolism. Cochrane Database Syst. Rev..

[10] Kaymaz C., Tokgöz H.C., Kültürsay B., Hakgör A., Keskin B., Sekban A., Karagöz A. (2023). Current Insights for Catheter-Directed Therapies in Acute Pulmonary Embolism: Systematic Review and Our Single-Center Experience. Anatol. J. Cardiol..

[11] Jiménez D., Aujesky D., Moores L., Gómez V., Lobo J.L., Uresandi F., Otero R., Monreal M., Muriel A., Yusen R.D. (2010). Simplification of the pulmonary embolism severity index for prognostication in patients with acute symptomatic pulmonary embolism. Arch. Intern. Med..

[12] Sanchez O., Trinquart L., Caille V., Couturaud F., Pacouret G., Meneveau N., Verschuren F., Roy P.-M., Parent F., Righini M. (2010). Prognostic factors for pulmonary embolism: The prep study, a prospective multicenter cohort study. Am. J. Respir. Crit. Care Med..

[13] Mehran R., Rao S.V., Bhatt D.L., Gibson C.M., Caixeta A., Eikelboom J., Kaul S., Wiviott S.D., Menon V., Nikolsky E. (2011). Standardized bleeding definitions for cardiovascular clinical trials: A consensus report from the Bleeding Academic Research Consortium. Circulation.

[14] Lookstein R.A., Konstantinides S.V., Weinberg I., Dohad S.Y., Rosol Z., Kopeć G., Moriarty J.M., Parikh S.A., Holden A., Parikh S.A. (2026). Randomized controlled trial of mechanical thrombectomy with anticoagulation versus anticoagulation alone for acute intermediate-high risk pulmonary embolism: Primary outcomes from the STORM-PE trial. Circulation.

[15] Kuo W.T., Gould M.K., Louie J.D., Rosenberg J.K., Sze D.Y., Hofmann L.V. (2009). Catheter-directed therapy for the treatment of massive pulmonary embolism: Systematic review and meta-analysis of modern techniques. J. Vasc. Interv. Radiol..

[16] Sadeghipour P., Jenab Y., Moosavi J., Hosseini K., Mohebbi B., Hosseinsabet A., Chatterjee S., Pouraliakbar H., Shirani S., Shishehbor M.H. (2022). Catheter-Directed Thrombolysis vs Anticoagulation in Patients With Acute Intermediate-High-risk Pulmonary Embolism: The CANARY Randomized Clinical Trial. JAMA Cardiol..

[17] Xing X., Liu J., Deng Y., Xu S., Wei L., Yang M., He X., Cao B., Huang X., Yue Q. (2020). Impact of renal function on the prognosis of acute pulmonary embolism patients: A systematic review and meta-analysis. Expert Rev. Respir. Med..

[18] den Exter P.L., van der Hulle T., Lankeit M., Huisman M.V., Klok F.A. (2013). Long-term clinical course of acute pulmonary embolism. Blood Rev..

[19] Elmewafy A.T., Waller J., Alaguraja P., Desor K., Antoun I. (2025). The Prognostic Value of Troponin in Acute Pulmonary Embolism: A Systematic Review and Meta-Analysis. Catheter. Cardiovasc. Interv..

[20] Peracaula M., Sebastian L., Francisco I., Vilaplana M.B., Rodríguez-Chiaradía D.A., Tura-Ceide O. (2024). Decoding Pulmonary Embolism: Pathophysiology, Diagnosis, and Treatment. Biomedicines.

[21] Donzé J., Labarère J., Méan M., Jiménez D., Aujesky D. (2011). Prognostic importance of anaemia in patients with acute pulmonary embolism. Thromb. Haemost..

