# Supplementary material for: Predictors of 90-Day Mortality and the Association of Reperfusion Therapy with 90-Day Mortality in Intermediate-High and High-Risk Pulmonary Embolism: A Real-World Multidisciplinary Cohort Study
Source: Life (Basel). 2026 Jul 17;16(7):1189. doi: 10.3390/life16071189 (PMC13412475; doi:10.3390/life16071189)
Supplement: Supplementary file 1 [file life-16-01189-s001.zip › life-4403886-supplementary.pdf]

Supplementary Table S1. History of Thromboembolic Events and Risk Factors in the Study Population

Risk Factor	N	%
Previous pulmonary embolism	4	(4)
Previous deep vein thrombosis	7	(6)
Prolonged immobilization	30	(26)
Trauma	12	(11)
Surgery within the last 3 months	28	(25)
Pregnancy / postpartum (n=62 women)	1	(2)
Oral contraceptive use (n=62 women)	2	(3)
Malignancy	16	(14)

Supplementary Table S2. Clinical and Laboratory Findings at Diagnosis

Characteristic	Mean ± SD / n	% / Median (Min–Max)
**sPESI score**		
0	7	(6)
1	33	(29)
2	45	(40)
3	26	(23)
4	2	(2)
5	1	(1)
**30-day mortality risk**		
Intermediate-high	98	(86)
High	16	(14)
**Diagnostic modality**		
CT pulmonary angiography	96	(84)
V/Q scintigraphy	18	(16)
**Right ventricular dilatation at diagnosis**	114	(100)
D-dimer, µg/L	254 ± 581	109 (7-5403)
Pro-BNP, pg/mL	5218 ± 8869	437 (15-35000)
sPAP, mmHg	44 ± 11	45 (20-75)
Hemoglobin, g/dL	12.0 ± 2.1	12.1 (6.5-16.9)
Platelets, $\times 10^3/\mu L$	242 ± 103	220 (30-629)
aPTT, seconds	28.60 ± 5.21	27.45 (20.00-46.60)
Creatinine, mg/dL	1.27 ± 0.90	1.03 (0.17-7.84)
ALT, U/L	61 ± 115	23 (5-757)

Abbreviations: sPESI, simplified Pulmonary Embolism Severity Index; V/Q, ventilation/perfusion; sPAP, systolic pulmonary artery pressure; aPTT, activated partial thromboplastin time; ALT, alanine aminotransferase; SD, standard deviation.

Supplementary Table S3. Risk Stratification According to Treatment Strategy

Risk group	Anticoagulation n (%)	Systemic thrombolysis n (%)	Catheter-directed therapy n (%)
Intermediate-high risk (n=98)	77 (78.6%)	13 (13.3%)	8 (8.2%)
High risk (n=16)	6 (37.5%)	9 (56.2%)	1 (6.2%)

Supplementary Table S4. 90-day Mortality by Risk Group and Treatment Strategy

Risk group	Anticoagulation deaths/total (%)	Reperfusion* deaths/total (%)	p-value
Intermediate-high	21/77 (27.3%)	0/21 (0.0%)	0.005
High risk	5/6 (83.3%)	3/10 (30.0%)	0.119

*Reperfusion = systemic thrombolysis or catheter-directed/EKOS

Suplementary Table S5. ROC Analysis of Variables Predicting 90-Day Mortality

Variable	AUC (95% GA)	p-value	Youden Index	Cut-Off	Sensitivity (%)	Specificity (%)
sPESI	0.744 (0.654 - 0.821)	**<.001**	0.399	>2	55	85
Age	0.667 (0.573 - 0.753)	**.003**	0.333	>66	86	47
Hemoglobin	0.685 (0.591 - 0.769)	**.001**	0.395	≤11.7 g/dL	72	67
Smoking (pack-years) (n=49)	0.697 (0.549 - 0.820)	**.021**	0.32	>47	47	85
BMI	0.618 (0.523 - 0.708)	.059	0.196	≤28.1 kg/m²	66	54
Pro-BNP	0.605 (0.510 - 0.696)	.109	0.341	>5480 pg/mL	52	82

Abbreviations: AUC, area under the curve; ROC, receiver operating characteristic; sPESI, simplified Pulmonary Embolism Severity Index; BMI, body mass index; Pro-BNP, pro–B-type natriuretic peptide.

Supplementary Table S6. Univariate Analysis of Factors Associated with 90-Day Mortality

Factor	Category	Mortality (+) (n=29) n (%)	Mortality (–) (n=85) n (%)	p-value	OR (95% CI)
**Sex**	Male	16 (55%)	36 (42%)	.231	1.68 (0.72-3.92)
**Age**	>66	25 (86%)	45 (53%)	**.001**	**5.56 (1.78-17.34)**
**Smoking status**	Ex/Current	15 (52%)	34 (40%)	.271	1.61 (0.69-3.75)
**Smoking (pack-years) (n=49)**	>47	7 (47%)	5 (15%)	**.016**	**5.08 (1.27-20.36)**
**Prolonged immobilization**	Var	12 (41%)	18 (21%)	**.033**	**2.63 (1.06-6.49)**
**Trauma**	Var	2 (7%)	10 (12%)	.461	0.56 (0.11-2.70)
**Surgery (last 3 months)**	Var	6 (21%)	22 (26%)	.575	0.75 (0.27-2.07)
**History of malignancy**	Var	4 (14%)	12 (14%)	.965	0.97 (0.29-3.29)

OR: Odds Ratio; CI: Confidence Interval.

Supplementary Table S7. Comorbidities and Associated Conditions in Relation to 90-Day Mortality

Factor	Mortality(+) (n=29) n (%)	Mortality(–) (n=85) n (%)	p-value	OR (95% CI)
**History of cerebrovascular event**	7 (24%)	5 (6%)	**.006**	5.09 (1.47-17.61)
**Chronic kidney disease**	8 (28%)	8 (9%)	**.015**	3.67 (1.23-10.93)
**Heart failure / Coronary artery disease**	14 (48%)	27 (32%)	.110	2.00 (0.85-4.74)
**Diabetes mellitus**	12 (41%)	29 (34%)	.482	1.36 (0.57-3.24)
**Hypertension**	19 (66%)	52 (61%)	.677	1.21 (0.50-2.91)
**Atrial fibrillation**	7 (24%)	11 (13%)	.153	2.14 (0.74-6.18)
**Other comorbidities**	14 (48%)	47 (55%)	.513	0.75 (0.32-1.76)
**Presence of any comorbidity**	27 (93%)	73 (86%)	.306	2.22 (0.47-10.57)

OR: Odds Ratio; CI: Confidence Interval
